# Supplementary material for: Integrated Analysis of the Lung Microbiome and Metabolome Reveals Associations Between Amino Acid Metabolism and Pulmonary Fibrosis in a Bleomycin-Induced Mouse Model
Source: Int J Mol Sci. 2026 Jun 30;27(13):5895. doi: 10.3390/ijms27135895 (PMC13362081; doi:10.3390/ijms27135895)
Supplement: Supplementary file 1 [file ijms-27-05895-s001.zip › result/4.MetDiffAnalysis/B_28d.vs.C_28d/B_28d.vs.C_28d_all_cluster_heatmap_detail.pdf]

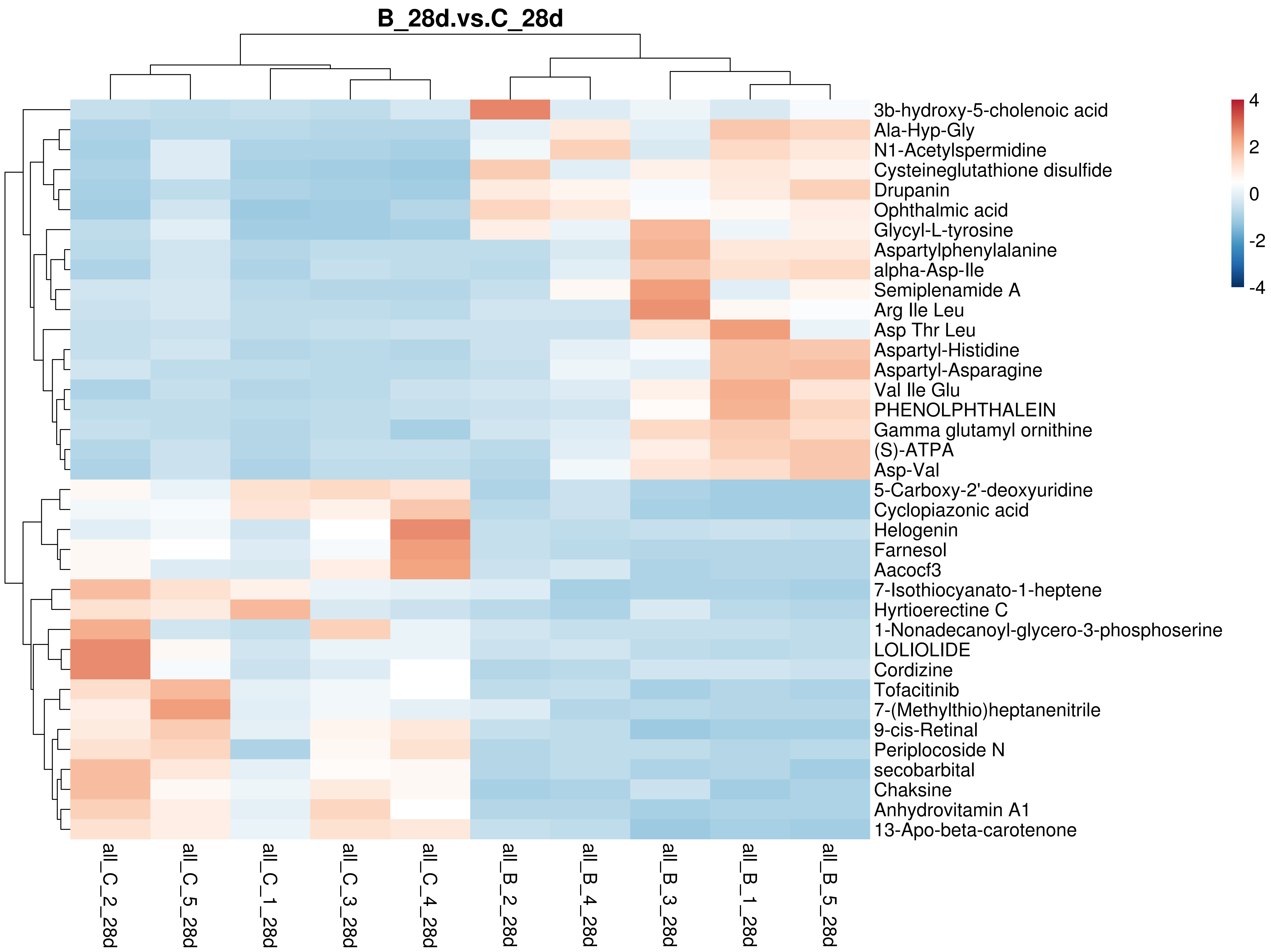

B_28d.vs.C_28d
3b-hydroxy-5-cholenoic acid
Ala-Hyp-Gly
N1-Acetylspermidine
Cysteineglutathione disulfide
Drupanin
Ophthalmic acid
Glycyl-L-tyrosine
Aspartylphenylalanine
alpha-Asp-Ile
Semiplenamide A
Arg Ile Leu
Asp Thr Leu
Aspartyl-Histidine
Aspartyl-Asparagine
Val Ile Glu
PHENOLPHTHALEIN
Gamma glutamyl ornithine
(S)-ATPA
Asp-Val
5-Carboxy-2'-deoxyuridine
Cyclopiazonic acid
Helogenin
Farnesol
Aacocf3
7-Isothiocyanato-1-heptene
Hyrtioerectine C
1-Nonadecanoyl-glycero-3-phosphoserine
LOLIOLIDE
Cordizine
Tofacitinib
7-(Methylthio)heptanenitrile
9-cis-Retinal
Periplocoside N
secobarbital
Chaksine
Anhydrovitamin A1
13-Apo-beta-carotenone
all_C_2_28d
all_C_5_28d
all_C_1_28d
all_C_3_28d
all_C_4_28d
all_B_2_28d
all_B_4_28d
all_B_3_28d
all_B_1_28d
all_B_5_28d
4
2
0
-2
-4
